# HapTree: A Novel Bayesian Framework for Single Individual Polyplotyping Using NGS Data

**DOI:** 10.1371/journal.pcbi.1003502

**Published:** 2014-03-27

**Authors:** Emily Berger, Deniz Yorukoglu, Jian Peng, Bonnie Berger

**Affiliations:** 1 Department of Mathematics, MIT, Cambridge, Massachusetts, United States of America; 2 Computer Science and Artificial Intelligence Laboratory, MIT, Cambridge, Massachusetts, United States of America; 3 Department of Mathematics, UC Berkeley, Berkeley, California, United States of America; Thomas Jefferson University, United States of America

## Abstract

As the more recent next-generation sequencing (NGS) technologies provide longer read sequences, the use of sequencing datasets for complete haplotype phasing is fast becoming a reality, allowing haplotype reconstruction of a single sequenced genome. Nearly all previous haplotype reconstruction studies have focused on diploid genomes and are rarely scalable to genomes with higher ploidy. Yet computational investigations into polyploid genomes carry great importance, impacting plant, yeast and fish genomics, as well as the studies of the evolution of modern-day eukaryotes and (epi)genetic interactions between copies of genes. In this paper, we describe a novel maximum-likelihood estimation framework, HapTree, for polyploid haplotype assembly of an individual genome using NGS read datasets. We evaluate the performance of HapTree on simulated polyploid sequencing read data modeled after Illumina sequencing technologies. For triploid and higher ploidy genomes, we demonstrate that HapTree substantially improves haplotype assembly accuracy and efficiency over the state-of-the-art; moreover, HapTree is the first scalable polyplotyping method for higher ploidy. As a proof of concept, we also test our method on real sequencing data from NA12878 (1000 Genomes Project) and evaluate the quality of assembled haplotypes with respect to trio-based diplotype annotation as the ground truth. The results indicate that HapTree significantly improves the switch accuracy within phased haplotype blocks as compared to existing haplotype assembly methods, while producing comparable minimum error correction (MEC) values. A summary of this paper appears in the proceedings of the RECOMB 2014 conference, April 2–5.

## Author Summary

While human and other eukaryotic genomes typically contain two copies of every chromosome, plants, yeast and fish such as salmon can have strictly more than two copies of each chromosome. By running standard genotype calling tools, it is possible to accurately identify the number of “wild type” and “mutant” alleles (A, C, G, or T) for each single-nucleotide polymorphism (SNP) site. However, in the case of two heterozygous SNP sites, genotype calling tools cannot determine whether “mutant” alleles from different SNP loci are on the same or different chromosomes. While the former would be healthy, in many cases the latter can cause loss of function; it is therefore necessary to identify the phase—the copies of a chromosome on which the mutant alleles occur—in addition to the genotype. This necessitates efficient algorithms to obtain accurate and comprehensive phase information directly from the next-generation-sequencing read data in higher ploidy species. We introduce an efficient statistical method for this task and show that our method significantly outperforms previous ones, in both accuracy and speed, for phasing triploid and higher ploidy genomes. Our method performs well on human diploid genomes as well, as demonstrated by our improved phasing of the well known NA12878 (1000 Genomes Project).

This Methods article is associated with RECOMB 2014.

## Introduction

**Figure 1:**
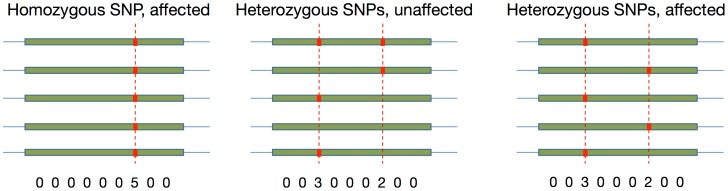
Loss of function in different polyplotypes of a sample pentaploid genome. As the loss of function is often determined by whether a healthy copy of a gene exists, knowing the genotype vector is sufficient if there is a single SNP site. In the case of two SNP sites however, the genotype vector cannot be used to unambiguously determine loss of function, and phasing is required.

While human and other eukaryotic genomes typically contain two copies of every chromosome, plants, yeast and fish such as salmon can have strictly more than two copies of each chromosome. By running standard genotype calling tools, it is possible to accurately identify the number of “wild type” and “mutant” alleles (A, C, G, or T) for each single-nucleotide polymorphism (SNP) site. However, in the case of two heterozygous SNP sites, genotype calling tools cannot determine whether “mutant” alleles from different SNP loci are on the same or different chromosomes (i.e. compound heterozygote). While the former would be healthy, in many cases the latter can cause loss of function; it is therefore necessary to identify the phase (*phasing*) —the copies of a chromosome on which the mutant alleles occur—in addition to the genotype (Figure 1). This necessitates efficient algorithms to obtain accurate and comprehensive phase information directly from the next-generation-sequencing read data in higher ploidy species.

Various sources of information can be utilized for the computational identification of an individual's diplotype/polyplotype: pedigree (e.g. trio-based phasing) [1]–[3], population structure of variants (e.g. phasing by linkage disequilibrium) [3]–[6] and more recently by identity-by-descent in unrelated individuals [7], [8], as well as sequencing read datasets [9]–[13]. Among these approaches, methods for sequence-based haplotype phasing are the only viable approach for haplotype phasing on a single individual member of a species (assuming homologous chromosomes are sequenced together), as other approaches either require family members or a population. For an individual diploid genome, the problem of reconstructing the diplotype using sequence information, the diploid phasing problem, is equivalent to the identification of the sequence of alleles on either parental haplotype. If this sequence is correctly inferred, then the other haplotype will automatically carry the corresponding opposite alleles (reference or alternative). Solving an error-free version of the diploid haplotype reconstruction problem is straightforward: the haplotype of each connected (by reads) component of heterozygous SNPs can be obtained by propagating allele information within reads. In reality, however, sequencing errors as well as false read mappings cause conflicts within sequence information, requiring a mathematical formulation of the haplotype reconstruction problem. Among various formulations suggested for this problem, the most commonly used is an NP-hard minimum error correction (MEC) definition [14], [15], which aims to identify the smallest set of nucleotide changes required within mapped fragments that would allow a conflict-free separation of reads into two separate homologous chromosomes (or a bipartite separation of the fragment conflict graph). Some of the solutions proposed for this problem include: HapCUT[9], an algorithm for optimizing MEC score based on computing max-cuts of the fragment graph; Fast Hare [16], a heuristic that clusters reads into two sets in a greedy fashion, and HapCompass [10], a spanning tree based approach for minimizing fragment conflicts.

Unlike diploid genomes, computational identification of common chromosomal variants in polyploid genomes using sequencing data has received little attention, except in the pioneering work of Aguiar & Istrail [8]. Polyploidy studies are of importance as they allow a comprehensive investigation of variants within plant, fish, and yeast genomes and help understand mechanisms of eukaryotic evolution. However, haplotype reconstruction in polyploid genomes is fundamentally more complex, even in the error-free version of the problem (without sequencing errors or false read mappings). Due to the newness of the NGS-based biological research in polyploid genomes, the mathematical foundations of the polyploid phasing problem have not yet been established. The solution proposed by Aguiar & Istrail for single individual polyplotyping problem is based on phasing all possible SNP loci pairs independently while further consolidating this information in a separate stage in order to infer a set of haplotypes.

Diploid phasing methods focus on a given list of heterozygous variants that are guaranteed to contain a single reference allele, as well as an alternative allele (assuming all heterozygous loci are bi-allelic). In contrast, in the polyploid phasing problem, there is no such guarantee of a single type of heterozygous SNP. Each heterozygous locus for a 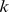-ploid chromosome can potentially contain from 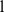 up to 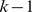 alternative alleles within the heterozygous loci, significantly increasing the complexity of the phasing problem in comparison to the diploid case. Furthermore, in a diploid phasing setting, there are always two possible options for phasing a pair of SNP loci, regardless of what other SNPs they are phased with. These two options can be thought as parallel (alternative allele pairs and reference allele pairs are matched within themselves) or switched (each alternative allele is matched with the other reference allele). These two options are no longer relevant when the genome contains more than two copies of each chromosome, due to the fact that there are up to 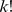 options when merging a phased haplotype block with another.

In this paper, we introduce a maximum-likelihood formulation of the polyploid full haplotype reconstruction problem and present a haplotype assembly algorithm, HapTree, which concurrently performs SNP-pair phasing and full haplotype assembly based on a probabilistic framework. We observe that, on simulated polyploid data, HapTree substantially improves the phasing capabilities and performance of any existing program. Because real polyploid data is hard to come by, we also evaluate HapTree on real human diploid data and find that, when compared to the more accurate trio-based data as the ground truth [17], HapTree significantly reduces the number of switch errors, while remaining on par in terms of MEC score over existing single-individual haplotype assembly methods for diploid genomes. We also introduce a relative likelihood (RL) score definition for annotation-free evaluation of phasing quality for polyploid haplotype assembly as an alternative to MEC score. Using simulated polyploid sequencing datasets, we demonstrate that RL-score performs significantly better at capturing haplotype assembly quality than MEC-score as ploidy increases.

## Method

### Overview of HapTree

The HapTree pipeline is designed to perform phasing and full haplotype assembly of a single genome. The key component of HapTree is a relative likelihood function which measures the concordance between the aligned read data and a given haplotype phase under a probabilistic model that also accounts for possible sequencing errors. To identify a phasing solution of maximal likelihood, HapTree finds a collection of high-likelihood solutions for phases of the first 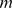 SNP loci and extends those to high likelihood phases of the first 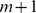 SNP loci, for each incremental 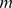. In each step, HapTree maintains only the set of likely partial phases to be extended in next steps. Finally, a phase of maximal likelihood for all loci is obtained after the extension of the last SNP locus.

Broadly speaking, HapTree aims to discover the best, or *maximum likelihood*, haplotype based on the read data available. Theoretically, one could enumerate all possible haplotypes, compute the likelihood of each being the true haplotype (using formulas described below), and choose the most likely one; in most cases this approach is intractable as there are exponentially many possible haplotypes. HapTree therefore has a variety of ways of trimming down the solution set from all possible haplotypes to a much smaller set of more likely solutions, making the problem tractable. It does so by taking an inductive approach, generating a collection of *likely* phasing solutions for the first two SNPs in the genome, and then extending those to phasing solutions of the first three SNPs, and those to the first four SNPs, and so on. When extending any particular solution, HapTree chooses (based on computing likelihoods) how the alleles of the newly added SNP may be assigned to chromosomes; it includes only those assignments that are sufficiently likely. Additionally, if HapTree finds after extending all solutions to include the next SNP that there are too many *likely* solutions, it throws the worst (*least likely*) solutions away. Upon including all SNPs to be phased, HapTree randomly chooses a solution of maximum likelihood from amongst the solutions it has found.

### Availability

An implementation of our method, HapTree, is available for download at: http://groups.csail.mit.edu/cb/haptree/

### Definitions and Notation

We describe below the problem of sequence-based polyploid haplotype assembly and provide basic technical notation that will be useful for describing our method. We assume for now that each SNP locus to be phased is bi-allelic (i.e. contains only two possible alleles, one being the reference allele). We further assume that for each SNP locus 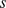, the genotype of 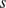 is known and is defined to be the number of chromosomes carrying the alternative allele (denoted by 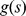). If 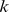 denotes the ploidy, 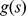 can range from 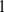 to 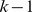 for heterozygous loci 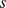. At this point, we would like to note that these two assumptions are made for the sake of simplicity of method description and implementation, though the genotype information does tend to be available. After describing our method we also describe the changes needed to our original approach to accommodate multi-allelic and genotype-oblivious polyploid haplotype assembly. At this time our implementation accommodates the aforementioned simpler case of bi-allelic SNPs and known genotypes; it is simple to extend this implementation to the more general case, and we describe such an extension in **Discussion**.

We denote the sequence of observed nucleotides of a fragment simply as a “read” (independent from single/paired-end reads and sub-reads of a strobe read structure). The set of all reads is denoted as 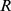. We define a read 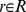 as a vector with entries 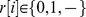 where a 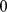 denotes the reference allele, a 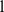 the alternative allele, and a 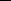 indicates one of two possibilities: First, that the read does not overlap with the corresponding SNP locus, or second, that neither the reference nor alternative allele is present and hence there must be a read error. A read 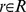 *contains* a SNP 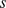 if 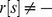. A read can also be represented as a dictionary or mapping with keys the positions (from amongst the SNPs to be phased) of SNP loci it contains and values of either reference allele or alternative allele, represented by 0 and 1 respectively (e.g. 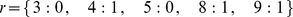). As current sequencing technologies generate read data with a certain rate of sequencing errors, some of the positions within a read likely contain false nucleotide information. Among these erroneous bases, unless they are located at SNP loci and contain opposite allele information, we ignore them by representing them with 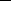, and thus keep only confounding sequencing errors that can affect phased haplotype results. For each read 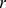 and for each SNP locus 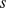, we assume an error rate of 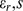 and a probability of opposite false allele information 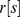 is equal to 
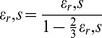
. We modify this error rate by a factor of two-thirds because conditional on there being an error, we model the error as equally likely to be any of the three other alleles. Two of the three of these alleles are neither the reference nor the alternative allele and thus we know that an error has been made in this case. Therefore, two-thirds of the time the erroneous alleles produced are known as such and may be thrown out, leaving a true error only one-third of the time. We represent these error rates as matrices 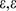. At this time our method assumes uniform error rates with respect to the SNP position; the error rate is supplied by the user and ought to depend on the read sequencing technologies used.

Upon the set of SNP loci 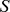 and read set 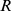; we define a *Read Graph*, 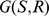, such that there is a vertex for each SNP locus 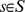 and an edge between any two vertices 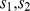 if there is some read containing both 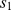 and 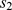; equivalently if 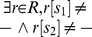. Without loss of generality, we assume that 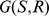 is connected; otherwise each connected component can be processed independently.

#### Vector set

A *k-ploidy phase* of 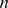 SNPs with genotypes 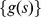 is a tuple of 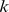 vectors (not necessarily distinct) 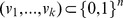 satisfying the genotype allele counts property, that is: 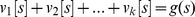 for all 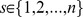. We will refer to this collection as a *vector set* and we think of each vector as a row vector.

We can build a phase by selecting a permutation of the alleles present for each SNP locus 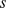. Note that the number of distinct permutations, 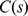, is strictly dependent on the genotype of the SNP and in the diploid bi-allelic case is equivalent to selecting the chromosomes containing the alternative alleles, hence
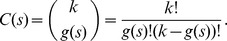



For example, let 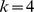, then 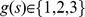. We enumerate the possible permutations below and include an example tetraploid genome.
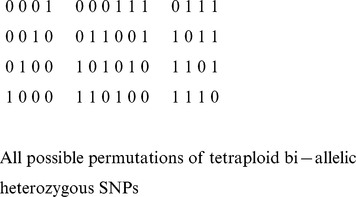





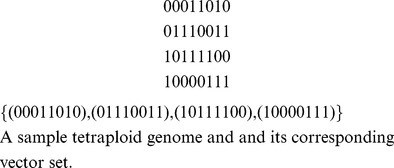



The sample tetraploid genome featured above on the right has a genotype vector: 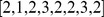; recall this counts the number of alternative alleles present at each SNP site. For any SNP 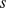, let 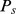 denote the set of distinct allele permutations at SNP locus 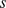. Throughout we are indifferent to the order of each chromosome, with this in mind we can see that the total number of phases is bounded below by 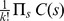.

#### Likelihood of a phase

We formulate the haplotype reconstruction problem as identifying the most likely phase(s) given the read data 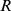, all SNP loci 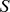, as well as their genotypes, and sequencing error rates 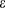. We assume the sequencing errors are independent of each other, that is for all 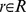 and all 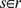, that 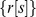 are independently correct with probabilities (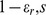) and incorrect with probabilities 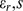. Let 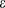 be a matrix containing all of these probabilities: 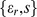. Given a vector set, 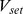, corresponding to a phase, 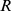, and 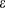; the likelihood of the phase is determined by:
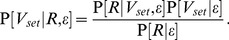
(1)


As 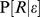 depends only on 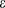 and the read set 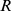, it is therefore the same across all vector sets. Hence, we define a *relative likelihood* measure (RL) as
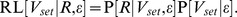



As for 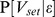, there are several ways this can be modeled depending on the situation. For polyploid simulated data, we can assume that 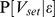 is equal for almost all vector sets, excluding ones containing duplicate vectors. Let 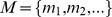 be the set of the multiplicities in 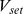; for example, if 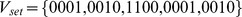 then 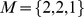. The probabilities 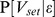 will differ multiplicatively by multinomial coefficients 
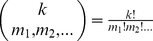
. Specifically:
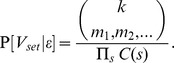



For real diploid data, there will never be duplicate vectors. To model 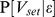, we might assume that since mutations tend to occur together, adjacent SNP sites are more likely to be phased in parallel 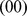 or 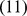 than switched 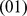 or 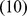. Let 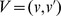 and let 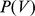 denote the number of adjacent SNPs that are parallel in 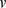 and 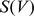 the number of adjacent SNPs that are switched in 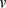 (we must only consider 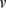 as it determines 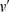). For example, if 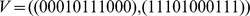, then 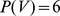 and 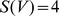. For some 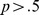 (denoted as *parallel bias*) and 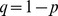, we model this vector set probability as
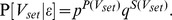



Finally, we consider 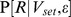. For a given 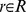 and 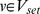, let 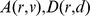 denote the positions of SNP loci where 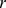 and 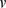 agree and disagree respectively. For example, if 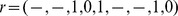 and 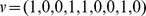, then 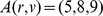 and 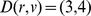 We may now compute the desired probability, that is:
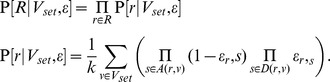



The goal of our haplotype reconstruction problem is to find the vector set(s) maximizing the product 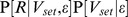, equivalently 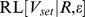. However, as the number of possible phases is on the order 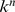, checking all of these is intractable. Our solution is based on finding high likelihood phases for the first 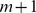 SNPs, conditioned on a collection of high likelihood phases for the first 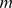 SNPs.

#### Semi-reads and sub-reads

To properly describe our method we must first define the *semi-reads* of a SNP locus 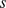 and the *sub-reads* of a subset 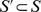. 

Semi-reads. To form the set of *semi-reads* of 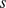, denoted 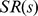, include each read 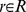 that contains both 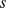 and some 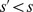 (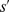 is upstream of 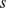) and ignore all information from 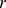 on SNPs 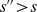 (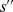 is downstream of 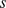). Suppose the set of reads is:

{1∶1, 2∶1, 3∶1, 4∶1} {3∶1, 4∶1, 5∶0, 6∶0} {4∶0, 5∶1, 6∶1} {4∶0, 5∶1, 6∶1, 7∶0} {5∶0, 6∶0, 7∶1} {5∶1, 6∶1, 7∶0}

The corresponding semi-reads for each SNP locus would be:


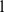
 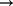 None 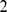 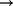 {1∶1, 2∶1} 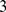 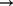 {1∶1, 2∶1, 3∶1} 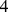 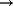 {1∶1, 2∶1, 3∶1, 4∶1} {3∶1, 4∶1}


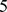
 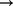 {3∶1, 4∶1, 5∶0} {4∶0, 5∶1} {4∶0, 5∶1}


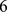
 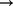 {3∶1, 4∶1, 5∶0, 6∶0} {4∶0, 5∶1, 6∶1} {4∶0, 5∶1, 6∶1} {5∶0, 6∶0} {5∶1, 6∶1}


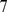
 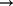 {4∶0, 5∶1, 6∶1, 7∶0} {5∶0, 6∶0, 7∶1} {5∶1, 6∶1, 7∶0}

Sub-reads. The *sub-reads* of 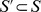, denoted 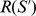, are obtained by, for each 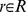, removing all keys 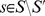 to form 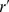, and then adding 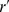 to 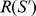 if the length of 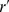 is at least 2. Alternatively, 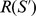 corresponds to the set of reads relevant to the problem of only phasing 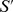. Continuing with the example above, if 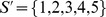, then
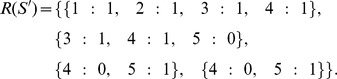



### HapTree

Our main approach to solving the single individual polyploid haplotype assembly problem is by finding highly probable solutions on 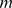 SNPs and extending those to highly probable solutions on 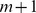 SNPs. Our algorithm has two fundamental parts: branching and pruning. For each connected component of the 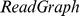, 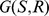, we inductively generate a collection of high likelihood phases on the first 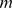 SNPs. For each of these phases, we branch them to phases on 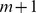 SNPs by considering all possible orderings of alleles for position 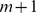 and including branches for those which occur with probability above a certain threshold. After doing so, we prune the tree of phases by removing all leaves that occur with probability sufficiently less than the most probable leaf. We discuss both parts in more detail below. We note that although a dynamic programming algorithm can be directly applied to infer the best solutions under HapTree's likelihood model, we instead developed HapTree, which is substantially faster than exact dynamic programming but with nearly identical empirical performance.

#### Extension

We first describe how to extend an existing a haplotype assembly 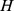 on 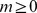 SNPs onto the 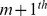 SNP 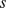. Recall the set of permutations of 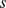 is denoted 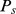 and one particular permutation as 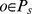. An extension 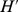 of 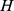 onto SNP locus 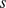 can be defined by appending some permutation 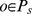 of alleles to 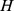; 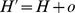. Note that it is possible for two distinct permutations to result in the same 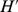: 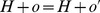. In these cases we do not include duplicates, as they are equivalent. Observe that if 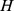 is empty, all allele permutations are the same as vector sets; we therefore include only one. For any 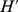, we can compute the probability of it being the correct haplotype (for the first 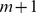 SNPs) conditioning on 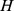 being correct (for the first 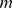 SNPs), as well as the semi-read data 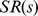 and error rate 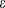. We express this below:
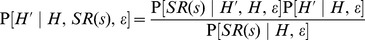
(2)


This computation is similar to those done above in equation (1). The EXTEND algorithm (Algorithm 1) is given below, which returns a list of all extensions 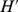 of 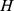 that occur with probability above a certain threshold, 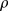, given haplotype 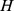.

#### Branching

Here we define *branching* a collection of haplotypes 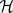 with threshold 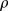 to SNP 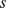: BRANCH(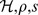) (Algorithm 2). We assume all 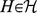 phase the first 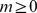 SNPs and that SNP 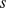 is the 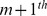 SNP. The act of branching 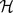 returns 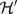: a list of all extensions generated by EXTEND with threshold 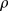 for all 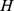 in 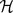. To initialize BRANCH we EXTEND the empty vector set to an arbitrary permutation of the alleles of the first SNP, as all permutations are equivalent as vector sets.

#### Pruning

For a collection of haplotypes 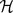 of SNPs 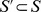, we can compute the relative likelihood of each haplotype conditioned on the sub-reads 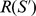 and error rate 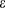; we write this as 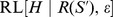. The same computation as performed in equation 1 yields:
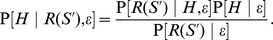



Since 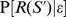 does not depend on 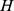:
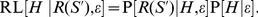
(3)


The goal of PRUNE(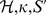) (Algorithm 3) is to return a subset 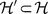 containing only sufficiently probable haplotypes. It does so by computing the relative likelihood of the most probable 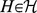, that is 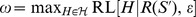, and adding 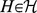 to 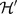 if 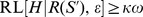, where 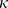 is between 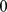 and 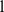. We note that that one can compute 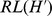 from 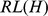 by only looking at the semi-reads 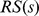: we store the relative likelihood values for all 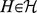 and update them when branching to 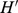; PRUNE is therefore no more costly than BRANCH.

#### Main algorithm

Here we give a high-level description of our overall haplotype assembly method HapTree(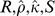) (Algorithm 4) using the EXTEND, BRANCH, and PRUNE algorithms. We generate high likelihood phases for the first 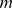 SNPs, BRANCH those phases to include 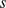 (the 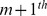 SNP), then PRUNE the resulting phases, and repeat for 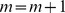. We begin with an arbitrary permutation of the first SNP, since all orderings result in the same vector set. For the final step, we PRUNE with 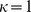, and therefore return only the maximally probable phases that we have found; if this set is of size greater than one, we choose a phasing from within it randomly. More generally, below we take 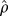 and 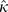 to be vectors, as 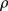 and 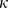 may depend on 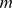, the size of 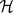 or other user-specified variables.

## Results

### Scoring and Evaluation

Determining the quality of a phasing solution depends on whether the true phase is known. When no such information is avaliable, the Minimum Error Correction (MEC) score [15] is a widely used scoring function to measure the quality of phasing solutions. The MEC score is defined as the minimum (amongst chromosomes) number of mismatches between a phase 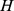 and the read set 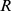. A number of existing programs, including HapCut [9], find phasing solutions by optimizing the MEC score in diploid cases. For higher ploidy the MEC score can no longer be reliably used because unlike in the diploid case, the phase of any one chromosome does not determine the phases of the others. Moreover, the MEC score does not distinguish between two separate phases of a pair of SNP loci with different non-zero counts of 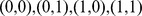 in their vector sets. Finally, unlike in the diploid case, a phase of a pair of SNP loci containing a set of parallel alleles does not prevent it from containing a set of switched alleles as well. To demonstrate these issues, consider two possible vector sets corresponding to phases of a pair of triploid SNPs both with genotype 2: 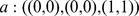 and 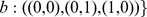. If the read data is 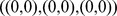, it is clear from a probabilistic standpoint that phase 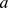 is a better fit, but both 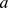 and 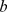 have equal MEC scores. This effect is exaggerated as 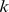 increases.

**Figure 2:**
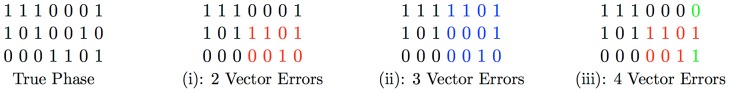
Examples of *Vector Error* in a sample tetraploid genome; the true phase is on the left and examples with two, three, and four vector errors are on the right.

When a true phase is available, there are a variety ways to evaluate how accurate any predicted phase is. A widely used measure in diploid phasing is switch error, which is calculated as the number of positions where the two chromosomes of a proposed phase must be switched in order to agree with the true phase. For polyploid phasing, we generalize switch error to *vector error*. In higher ploidy cases, at any SNP locus, it is possible for no chromosomes in a proposed phase to require a switch or anywhere from 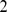 to 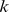 chromosomes to require switches, in order for a proposed phase to agree with the true phase. We do not wish to penalize a solution where only two vectors must be switched at a given position with the same penalty to be used for a solution in which all vectors must be switched. The *vector error* of a proposed phase (with respect to the true phase) is defined by the minimum number of segments on all chromosomes for which a switch must occur; for the diploid case this score is exactly twice the switch error. One may also think of the vector error as the minimum number of segments a proposed phase and the true phase have in common, less the ploidy. Even for triploid genomes, the vector error is more discriminative than switch error. Consider the following example in Figure 2:

In Figure 2 phase (i) is a more accurate phase than (ii), and phase (ii) more accurate than phase (iii). The segments are broken up by row and color: phase (i) having five segments, phase (ii) having six, and phase (iii) having seven. Note that there may be several ways to break a vector set into a minimal number of segments; phase (ii) is such an example. Finally, we remark that vector error can be computed in time 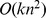, where 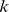 is the ploidy and 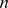 the block size.

### Results for Simulated Polyploid Data

#### Relative Likelihood (RL) objective function vs. MEC score for polyploid genomes

**Figure 3:**
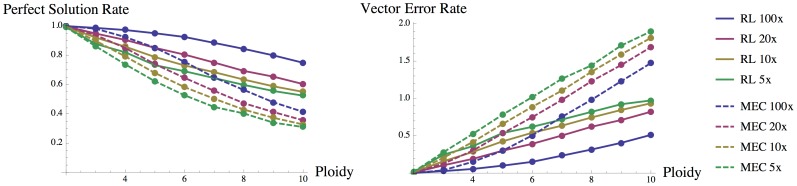
Proportion of perfectly phased SNP pairs and vector error rate for RL (solid line) and MEC (dashed line) optimization in 10000 trials over 5×, 10×, 20× and 100× coverage.

We assessed the effectiveness of our RL score by comparison to MEC score on simulated data. To do so, we simulated reads with error rate 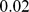 from a pair of phased 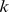-ploid SNP loci for different coverages (5×, 10×, 20×, 100×) and for 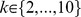. All possible phases were exhaustively enumerated, and phases of the maximal relative likelihood (RL) and phases of the minimal MEC score chosen. We computed the proportion of perfectly phased SNP pairs in both cases (perfect solution rate). Even with two SNP loci, RL significantly outperforms MEC for all 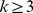 (Figure 3A). It is also worth noting that MEC (in comparison to RL) deteriorates more seriously in accuracy as ploidy 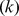 increases (Figure 3A). In addition, we also compared the vector error rate in both cases; for a pair of SNPs, this rate is the number of vectors from the proposed solution that cannot be matched with vectors from the true solution (Figure 3B).

The results demonstrate that the higher the ploidy, the better the relative likelihood (RL) score performs in comparison to MEC score for phasing a pair of SNPs (Figure 3). In fact, in simulations where 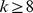, RL with 5× the coverage already outperforms MEC with 100× coverage. For the same coverage, RL always outperforms MEC for 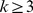, and they are equivalent in the diploid case 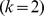.

#### Comparisons of HapTree and HapCompass

To evaluate the phasing capabilities of HapTree, we compare it with HapCompass [8] (latest version available at: www.brown.edu/Research/Istrail_Lab/hapcompass.php), to our knowledge the only other existing program that directly addresses polyploid haplotype assembly, over multiple depth coverage values and component sizes for triploid and tetraploid simulated genomes. We simulated triploid and tetraploid genomes with different block lengths (10, 20 or 40 SNP loci), different coverages (5×, 10×, 20× and 40×), SNP positions, and SNP densities. Throughout the simulations for both the triploid and tetraploid cases, our EXTEND module is run with threshold 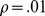 and PRUNE primarily with threshold 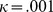. When the current number of haplotype options generated is above 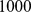, we prune more aggressively with 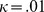 and when above 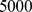, with 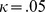. These parameters are chosen to ensure the efficiency of HapTree by only keep a tractable collection of promising solutions in each step. We also simulate a read set with uniform error rate and size dependent on coverage.

**Figure 4:**
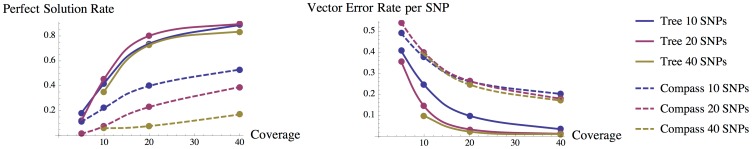
HapTree (solid lines) and HapCompass (dashed lines) on simulated triploid genomes: Likelihood of Perfect Solution and Vector Error Rates, 1000 Trials, Block lengths: 10, 20, and 40.

For the triploid case, we observed that HapTree finds a perfect solution at a rate independent of the number of SNPs used in the simulation; in contrast, HapCompass declines in performance the larger the block size (Figure 4). While both HapTree and HapCompass improve steadily the higher the coverage, in every case HapTree significantly outperforms HapCompass; the least significant improvement of 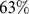 occurs in the case of 10 SNP loci and 10× coverage, whereas the most significant improvement occurs in the case of 40 SNP loci and 40× coverage. For both vector error rate and likelihood of perfect solution, we find that HapTree substantially outperforms HapCompass.

**Figure 5:**
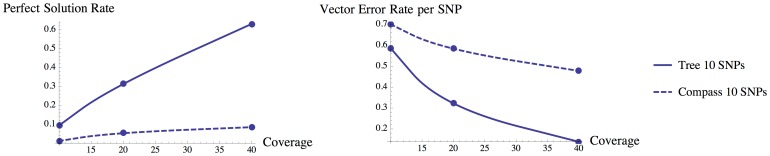
HapTree (solid line) and HapCompass (dashed line) on simulated tetraploid genomes: Likelihood of Perfect Solution and Vector Error Rates, 1000 Trials, Block length: 10.

For tetraploid simulations, HapTree significantly outperforms HapCompass with block length of 10 SNP loci (Figure 5). For larger block lengths HapCompass arrives at the perfect solution at a rate of less than 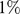; HapTree however does so at a rate between 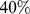 and 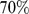 depending on block size and coverage at least 20×.

**Figure 6:**
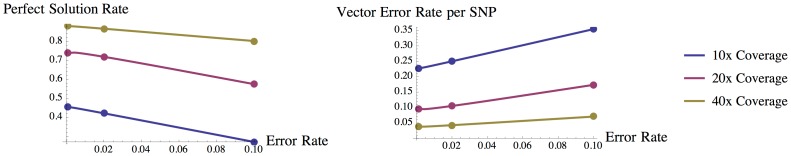
HapTree performance over varied error rates (.001, .02, .05, .1) and coverages (10×, 20×, 40×) on simulated triploid genomes: Likelihood of Perfect Solution and Vector Error Rates, 10000 Trials, Block length: 10.

We varied the allele error rates (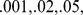 and 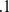) and observed decreases in accuracy that vary approximately linearly with the (uniform) allele error rates (Figure 6). The allele error rate is the likelihood of the sequencing technology to report the incorrect allele for a given position in one read. We ran 10000 trials for simulated triploid genomes of block size 10, with coverages 10×, 20×, and 40×.

For the simulations above in Figure 6, we modeled our read data on Illumina sequencing technologies; for more details, please see section **Simulated polyploid data generation** below. We also ran simulations on longer read data, modeled after 454 sequencing technologies and found almost identical results.

The primary reasons for HapTree's superior performance are, first, that HapTree's relative likelihood is more effective than HapCompass's MEC score (see **Relative likelihood vs MEC**); and second, that HapTree's inference algorithm is more accurate than the approximation algorithm used by HapCompass.

## Discussion

### Run-Time Evaluation

Not only does HapTree outperform HapCompass on phasing quality, it is also significantly faster, especially for longer block length. The median runtimes for block length 10 and 10× coverage were 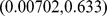 seconds for HapTree and HapCompass, respectively; for block length of 40 and 40× coverage, they were 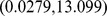 seconds, respectively.

### Results on Real Diploid Data

As seen in the results of Geraci et al. [18], there is no perfect solution for diploid phasing. HapCUT is one of the methods reported that consistently performs best or close-to-best for a variety of experiments. For a proof of concept of how HapTree would perform on real data, we ran HapTree and HapCUT using 454 and Illumina sequencing data of the well-studied NA12878 genome (1000 Genomes Project Phase 1) [17], and compared MEC scores as well as switch errors to a trio phasing annotation accepted as ground truth; we present these results in Table 1. The trio phasing annotation represents a high quality diplotype of NA12878 for all SNP sites where either parent (NA12891 or NA12892) is homozygous [17]. Note that we computed the number of switch errors within connected SNP components only, against SNPs whose phase has been determined by the trio-based phasing; we then sum over components. In this case, HapTree was run with a uniform error rate of 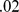, an EXTEND threshold 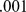, and primarily with a PRUNE threshold of 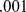. We begin to prune more aggressively when we have at least 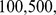 or 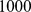 possible haplotypes with thresholds of 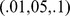 respectively. For the vector set prior, from examining the read data, we ran HapTree with parallel bias 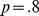.

**Table 1:** Results of switch error (switch) and MEC score for HapTree and HapCUT of whole-genome phasing using 454 and Illumina data.

Results	454		Illumina	
Method	MEC	Switch	MEC	Switch
HapTree	32818	**2978**	20339	**1888**
HapCUT	32781	3192	20290	1933

We found that HapTree and HapCUT perform almost identically in MEC scores, with HapCUT having marginally smaller scores for both 454 and Illumina data sets. It is worth noting that HapCUT optimizes MEC score, and MEC score measures only the consistency between a phasing solution and read data, not with the true phase.

Notably, when comparing to the ground-truth phase as determined by trio-based phasing, we found HapTree significantly outperforms HapCut in terms of switch error rate for the phasing experiments on the NA12878 genome for 454 and Illumina datasets. Although our method is not primarily designed for phasing diploid genomes, it is still able to achieve better phasing results, when compared to the state-of-the-art diploid method. Again, the results on real-world read datasets showed the superiority of our likelihood function over MEC score for NGS-based phasing.

### Simulated Polyploid Data Generation

#### Reads

To generate a paired-end read, we uniformly choose a starting point on the genome (we make sure the genome starts sufficiently before the first SNP and ends at the last). We fix the read-end length (read_len) to be 150. The fragment length (frag_len) is normally distributed with a mean of 550 and standard deviation of 30, but with min and max lengths of 500 and 600 respectively. The insert length (insert_len) is determined by the fragment length and read-end length, that is, insert_len  =  frag_len - 2read_len. Once we know the start and fragment length, we must choose from which chromosome to read; we do so uniformly from the 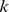 chromosomes. Finally, we add uniform error to the read; we choose a rate of 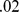, based on the reported error rate of Illumina sequencing technologies. For every SNP that the read covers, independently with probability 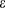 we flip the allele to any other allele; two-thirds of the time when we have this error, we can see that the allele present is neither the reference nor the alternative, and therefore we delete it. Hence, conditional on seeing a SNP in a read, it is incorrect with probability 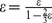 and correct with probability 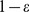.

#### Genomes

To simulate a genome, we fix a ploidy 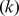 and the number of SNPs 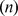. We determine the positions for the SNPs by randomly generating the distance between each pair of adjacent SNPs. We do so using a geometric random variable with parameter 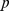 (SNP density); this choice is equivalent to assuming that any position is a SNP independently with probability 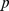. For phasing purposes, once one has generated the reads, the exact genomic positions are no longer relevant; they were only needed to simulate more accurate read data. We therefore refer to SNPs by their position amongst the SNPs, not their position in the genome. For each SNP, we randomly generate its haplotype, assuming for each chromosome, that the alternative and reference alleles are equally likely; if we generate a homozygous SNP, we try again. This procedure results in the likelihood of genotype 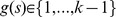 equal to 
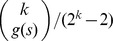
, and all orderings 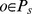 being equally likely. For the simulations discussed we use this model. Note, however, that HapTree is not dependent on this model. When running HapTree on real data, different assumptions ought to be made regarding the distributions of vector sets.

#### Coverage

For any genome, to generate a read set with 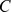
x coverage we need each base pair to be on average covered by 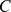 reads. To determine the number of reads to generate, we must know the length of the genome and the read length (read_len). The expected length of the genome is 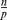 for SNP density 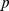, and the read_len is 150 for each end (of which there are two); therefore we simulate 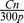 reads for 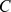
x coverage. Note that many of these reads will see only zero or one SNP(s), thus for 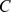
x coverage the number of useful reads for any SNP will be less than 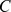.

## Discussion

We have presented a scalable algorithm, HapTree, for polyplotyping using NGS sequencing data and a new metric for measuring accuracy in this context. We have described an efficient algorithm to identify phases that maximize our RL metric, a relative likelihood function which measures the quality of a given phase according to the read dataset. We have demonstrated the advantages of such a likelihood formulation over the existing MEC score in phasing both polyploid and diploid genomes. HapTree not only substantially improves the efficiency and phasing accuracy of the state-of-the-art in polyploid phasing, but also produces more accurate phased haplotype blocks for diploid genomes, as compared to HapCUT, which is designed for diploid phasing by MEC score optimization. Our results indicate that HapTree can be used in phasing individual triploid and tetraploid genomes, as well as improving phasing of real diploid genomes. HapTree also easily scales to genomes of higher ploidy.

Our algorithm can be easily extended to phase data with multi-allelic SNPs and with unknown genotype information as well. With unknown genotype information and multi-allelic SNPs, instead of 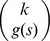 allele permutations, there are 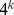 possibilities, since all 4 alleles (A,C,G,T) are possible for all 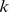 chromosomes. For bi-allelic SNPs with unknown genotypes, there are 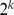, as all possible reference and alliterative allele permutations are allowable. Finally, when the genotype is known but a SNP is multi-allelic, we may use multinomial coefficients to compute the number of allele permutations allowable: 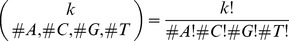, where 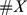 denotes the number of alleles 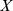 according to the genotype, where 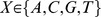. The only change to HapTree in these cases is that at each EXTEND step, we allow all allele permutation possibilities as dictated by whatever genotypic is available: we compute the probabilities for all 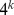, 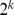, or 
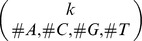
 possibilities (depending on the situation) as opposed to 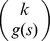 and EXTEND accordingly. Moreover, the type of information available does not need to be the same for all SNPs, since it only determines which allele permutations we introduce at the EXTEND step.

A future application of HapTree is genotype imputation, which can predict missing genotype from phasing results. As polyploid sequencing data becomes available, HapTree will be useful for the investigation of the role of heterozygosity in plant, fish, and other species. Moreover, accurate individual phases of diploid haplotypes can be assembled without the use of pedigree or population information.

A summary of this paper appears in the proceedings of the RECOMB 2014 conference, April 2–5 [19].
